# Novel Autosomal Recessive *c10orf2* Mutations Causing Infantile-Onset Spinocerebellar Ataxia

**DOI:** 10.1155/2012/303096

**Published:** 2012-08-11

**Authors:** Jessica N. Hartley, Frances A. Booth, Marc R. Del Bigio, Aizeddin A. Mhanni

**Affiliations:** ^1^Department of Biochemistry and Medical Genetics, University of Manitoba, Winnipeg, MB, Canada; ^2^Program of Genetics and Metabolism, Winnipeg, MB, Canada; ^3^Department of Pediatrics and Child Health, University of Manitoba, Winnipeg, MB, Canada; ^4^Department of Pathology, University of Manitoba, Winnipeg, MB, Canada

## Abstract

Recessive mutations in genes encoding mitochondrial DNA replication machinery lead to mitochondrial DNA depletion syndromes. This genetically and phenotypically heterogeneous group includes infantile onset spinocerebellar ataxia (OMIM# 271245) a neurodegenerative disease caused by mutations in the mtDNA helicase gene, *c10orf2*, with an increased frequency in the Finnish population due to a founder mutation. We describe a child of English descent who presented with a severe phenotype of IOSCA as a result of two-novel mutations in the *c10orf2* gene. This paper expands the phenotypic spectrum of IOSCA and adds further evidence for the presence of a genotype-phenotype correlation among patients with recessive mutations in this gene.

## 1. Introduction

Mitochondrial recessive ataxia syndromes caused by mutations in *c10orf2*, which encodes mitochondrial DNA (mtDNA) helicase, and in *POLG1*, which encodes mitochondrial DNA polymerase, can result in abnormal mtDNA replication leading to depletion in copy number. Mitochondrial DNA depletion results in a neurodegenerative course in infantile-onset spinocerebellar ataxia (IOSCA) (OMIM# 271245) caused by mutations in *c10orf2,* and in mitochondrial spinocerebellar ataxia epilepsy syndrome (MSCAE) (OMIM# 607459) and Alpers-Huttenlocher syndrome (OMIM# 203700) both caused by mutations in *POLG1* [1]. IOSCA is characterized by a period of normal development for approximately the first year of life followed by development of ataxia, hypotonia, loss of deep-tendon reflexes, and athetosis. Signs of advanced disease include ophthalmoplegia, sensorineural hearing loss, sensory axonal neuropathy, and epilepsy [2]. This condition was first described in Finland due to a homozygous founder mutation (1708A > G, Y508C) [3]. Other diseases causing mutations in the *c10orf2* gene have been reported subsequently [4–6]. Here we describe a child of English descent presenting with a phenotype of IOSCA who was found to be a compound heterozygote for two previously unreported presumed pathogenic mutations in the *c10orf2* gene.

## 2. Case Presentation

The propositus attained fine and gross motor milestones at appropriate ages until 8 months of age, at which time she began to regress. By 13 months she had lost her ability to control her head and could no longer sit independently or roll. She continued to achieve purposeful hand movements and was able to pick up small objects albeit with some difficulty. She was the first born to a healthy-27-year-old mother and a healthy-29-year-old father, both of English ancestry. The pregnancy was unremarkable with no history of exposure to teratogens. The perinatal and neonatal histories were unremarkable. Her birth weight was 3.615 kg (75th percentile), her length was 52.25 cm (75th percentile), and her OFC was 34.5 cm (50th percentile). Her Apgar scores were nine at one and at five minutes. The family history was unremarkable for neurodegenerative disease and consanguinity. 

The physical examination at 14 months revealed a nondysmorphic child. Her weight was 9.31 kg (40th percentile), height was 77 cm (50th percentile), and her OFC was 44.75 cm (2nd percentile). Cranial nerves examination was remarkable for disconjugate eye movements with inconsistent and variable nystagmus. Her fundus examination was normal. Her facial movements were symmetric. She had dyskinetic tongue movements and drooling. Her head control was fair and her muscle strength was within normal limits. She had significant hypotonia in both her trunk and limbs to passive movement but increased tone on vertical suspension. Her deep tendon reflexes were not elicitable and her plantar responses were flexor. She exhibited choreoathetoid movements in her limbs and marked truncal unsteadiness. 

Her neurodevelopmental status remained stable with no further regression over the course of the following year. At four years of age, she developed numerous clinical and electrographic seizures and subsequent epileptic encephalopathy. At this age her physical examination revealed the presence of ophthalmoplegia. 

Investigations including plasma, urine and cerebrospinal fluid amino acids, urine organic acids, plasma ammonia, serum carnitine levels, acylcarnitine profile, transferrin isoelectric focusing, lysosomal enzyme assay panel, plasma very long chain fatty acids, urine creatine and guanidinoacetate, screening for neuronal ceroid lipofuschinoses types 1 and 2, and screening for Niemann-Pick disease types A, B, and C failed to yield a diagnosis. Capillary lactate levels were inconsistently elevated, ranging from 0.6 to 5.3 mmol/L (normal range: 0.55–2.2), while the CSF lactate was normal. Serum alpha-fetoprotein was elevated ranging from 16 to 31 *μ*g/L (normal range: 0–7.0). Liver transaminases were normal. A karyotype analysis and molecular testing for Angelman syndrome, Rett syndrome, and ataxia with oculomotor apraxia type 2 were unrevealing. Respiratory chain enzyme analyses and histopathologic examination of muscle were nondiagnostic. Magnetic resonance imaging of the brain at 12 months of age was normal, but subsequent imaging showed increased prominence of the extra-axial spaces and evidence of atrophy. Nerve conduction studies revealed evidence of sensory neuropathy.

**Figure 1:**
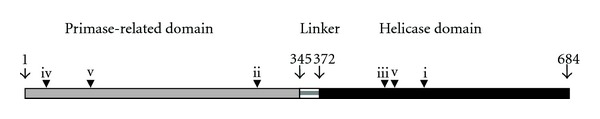
Schematic representation of the *c10orf2* gene showing the locations of previously reported recessive mutations: (i) Y508C [3], (ii) A318T [4], (iii) T451I [5], (iv) R29X [6], and (v) P83S/R463W [Current paper].

At age 5 years, her symptoms and signs worsened. Liver transaminases were elevated; AST was 280 *μ*/L (normal range 10–50) and ALT was 123 *μ*/L (normal range 4–30). Given the development of seizures, encephalopathy, ophthalmoplegia, intermittently elevated capillary lactate, and elevated liver transaminases, a mitochondrial DNA depletion syndrome was considered. *POLG1* mutation analysis was unrevealing. Sequencing of the *c10orf2* gene (Centogene GMBH) revealed two previously undescribed nucleotide changes, c.247C > T (P83S) and c.1387C > T (R463W). The P83S alteration occurs in the primase domain, and the R463W alteration occurs in the helicase domain (Figure 1). Both alterations occur in highly conserved regions and software analyses with PolyPhen [7], SIFT [8], and AGVGD [9] support their pathogenicity. The parents were shown to be carriers for one mutation each. 

**Figure 2:**
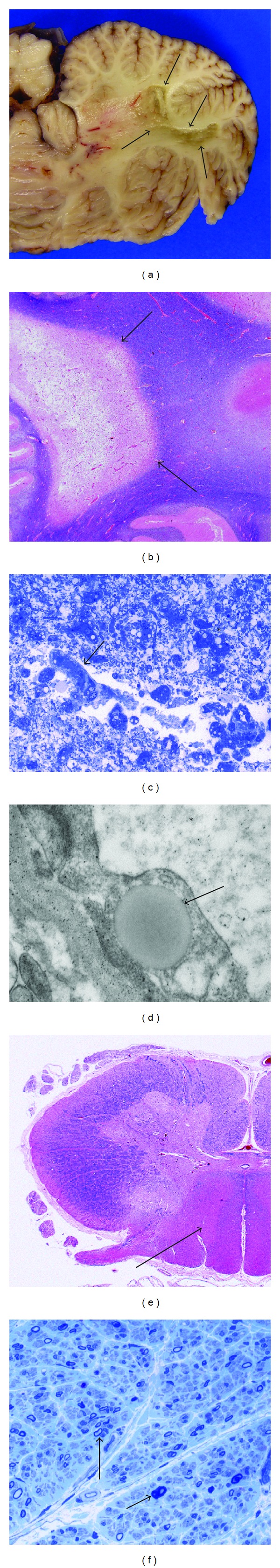
Pathologic findings. (a) Photograph of horizontal slice through right cerebellum and medulla showing irregular region of atrophy (arrows) in white matter lateral to the dentate nucleus. (b) Photomicrograph of cerebellum showing regional loss of myelin surrounding a cavity (arrows). (Solochrome cyanin/eosin stain; original magnification 12.5x.) (c) Photomicrograph of plastic-embedded sample of cerebellar lesion showing hypertrophic endothelium (arrow) and macrophages (*) with vacuolated cytoplasm. (Toluidine blue stain; original magnification 600x.) (d) Electron micrograph of capillary endothelium (*shows lumen) in frontal lobe showing lipid droplet (arrow) (original magnification 80000x). (e) Photomicrograph of spinal cord showing loss of myelin staining and atrophy of posterior columns (arrow). (Solochrome cyanin/eosin stain; original magnification 12.5x.) (f) Photomicrograph of plastic-embedded sample of sural nerve showing moderately severe loss of myelinated axons (residual axons appear as dark rings; arrows). (Toluidine blue stain; original magnification 600x.)

At 5 years of age, the child passed away due to bronchopneumonia. Autopsy revealed neurodegenerative disease characterized by mild cerebral atrophy, moderately severe neuronal loss in the cerebellar dentate nucleus, and inferior olivary nucleus of the medulla oblongata. Symmetric hypertrophic endothelial changes with reactive astroglial and microglial changes were present in the thalamus, superior, and inferior colliculi. The cerebellar white matter showed similar vascular changes and cavitation akin to those seen in Leigh's encephalopathy. Axons were lost from the posterior columns of spinal cord and posterior roots. Ultrastructural examination showed lipid droplets in the endothelial cells and moderately severe peripheral axon loss in the femoral and sural nerves (Figure 2). Skeletal muscle had secondary neurogenic changes and mild fibre type 2 atrophy. These neurodegenerative changes were considered to be attributable to a mitochondrial disorder. 

## 3. Discussion

The girl described herein presented at 8 months of age with a severe and progressive neurological phenotype suggestive of IOSCA and was shown to have two-novel mutations in the *c10orf2* gene; c.247C > T (P83S) and c. 1387C > T (R463W). The P83S alteration occurs in the primase domain (Figure 1) in a highly conserved position and is predicted to be pathogenic based on analyses with PolyPhen [7], SIFT [8], and AGVGD [9]. The R463W alteration occurs in the helicase domain (Figure 1) in a highly conserved position and software analyses have also suggested that it is likely pathogenic. The asymptomatic parents were each shown to harbor one of these mutations, adding further support to the pathogenicity.

Most reported recessive *c10orf2* mutations are the result of homozygosity for the Y508C Finnish mutation [3]. IOSCA however has also been reported in non-Finnish individuals due to other mutations including Y508C/A318T [4], Y508C/R29X [6], and T451I/T451I [5] (Figure 1). Whereas dominant mutations, causing progressive external ophthalmoplegia and mtDNA deletion tend to cluster in the linker region of the Twinkle protein, recessive mutations causing DNA depletion tend to cluster in the helicase or primase domains [10] (Figure 1). Our patient has two presumed pathogenic mutations, one in the primase domain of exon 1 and one in the helicase domain in exon 2. Recently, Goh and coworkers described a girl with mutations in the primase and helicase domains; she presented at 2 months of age with acute liver failure, abnormal neurologic examination, and Fanconi syndrome, and died at 6 months of age [6]. Considering that the primase domain's role is localizing the helicase to its target, we anticipate that mutations in this domain significantly impact the helicase function, hence resulting in a severe phenotype.

Lonnqvist and coworkers suggested that patients who are compound heterozygotes for Y508C or have two other pathogenic mutations have a severe phenotype compared to Y508C homozygotes [1]. These patients present around six months of age, rather than after one year of age and manifest abnormal liver transaminases [1, 4–6]. *In vivo *studies have shown abnormal helicase function in patients homozygous for T451I as compared to normal function in Y508C homozygotes [5] further demonstrating the severity of other genotypes.

The pathologic changes in our patient are not identical to any reported cases. The symmetric atrophic vascular hypertrophy changes in the thalamus and brainstem with pronounced cerebellar white matter involvement are histologically similar to those in Leigh's encephalopathy, but differ in localization. The abnormalities in the brain and spinal cord of previously reported children [11, 12] and one adult [13, 14] with *POLG1* mutations have some pathologic features similar to this patient. The dentate nucleus and posterior spinal cord changes of one child with two *c10orf2* mutations are also similar to our case [4]; however these changes do not appear to be pathognomonic for this condition [1, 6]. 

This paper further expands the phenotypic spectrum of *c10orf2* gene mutations and adds further evidence for the presence of a genotype-phenotype correlation among patients with IOSCA.
